# Supplementary material for: The urban carbon unlocking effect of digital infrastructure construction: A spatial difference-in-difference analysis from “Broadband China” pilot policy
Source: PLoS One. 2025 Jan 6;20(1):e0316202. doi: 10.1371/journal.pone.0316202 (PMC11703053; doi:10.1371/journal.pone.0316202)
Supplement: Attachment — Submitted filename: Response to Reviewers.docx [file pone.0316202.s001.docx]

**Response to Reviewers Comments**

Dear editor and reviewers:

On behalf of my co-authors, we thank you very much for your letter and for the reviewers’ comments concerning our manuscript entitled “**The urban carbon unlocking effect of digital infrastructure construction: A spatial difference-in-difference analysis from China (PONE-D-24-09583)**”. Those comments are all valuable and very helpful for revising and improving our paper, as well as the important guiding significance to our research. We have studied the comments carefully and have made a major revision and re-plan which we hope to meet with the approval. We tried our best to improve the manuscript and all changes made in the revised manuscript are **highlighted in blue** that will not influence the content and the framework of the paper. The point-by-point responds to reviewers’ comments are as follows:

**Reviewer # 1 comment:**

*1. This manuscript investigated the carbon lock-in phenomenon in the economic development of modern countries and how digital infrastructure construction addresses this issue. Taking the “Broadband China” policy as an example, this manuscript raises an interesting topic, which is of great significance to countries seeking structural transformation of their economies, especially developing countries like China.*

*However, it appears that the focus of this manuscript may have been misplaced. The manuscript in question examines the impact of BCP on CLI. While this issue is undoubtedly of great importance to China's economic development, such a narrow focus may diminish the academic merit of the study. This study would be more suitable for publication in a Chinese journal unless the authors decide to shift the focus of the study to reveal the impact of digital infrastructure on CLI and explore this phenomenon with China as the study area.*

Response 1: We would like to thank you for kindly reminding us. The revisions are as follows here for your convenience.

The research in this paper focuses on the impacts and mechanisms of BCP on city-level CLI. First, the findings provide a theoretical basis and practical examples for understanding the role of digital transformation in promoting low-carbon development. Second, the findings provide a quantitative assessment of policy effects for policymakers, which can help optimize and adjust existing policies for a more effective low-carbon transition. Further, the study emphasizes the impact of regional development differences on policy effectiveness, providing a new perspective for achieving coordinated regional development and balance. Finally, the study emphasizes the key role of technological progress and industrial structure upgrading in digital transformation. Most importantly, the results of the study add to the research on digital infrastructure and low carbon development, providing new ideas for low carbon transformation at the city and national levels, i.e., facilitating the optimization of economic structure and greening of energy consumption through digital technology facilities.

In fact, the Broadband China Strategy is one of China’s digital infrastructure construction projects. BCP’s goal is to build a high-speed, secure and reliable broadband network environment that supports and promotes the digital transformation and informatization development of the entire country through the construction and upgrading of broadband networks. Digital infrastructure construction includes various infrastructure elements, such as broadband networks, data centers, and cloud computing infrastructure. The Broadband China Strategy pays special attention to the construction and popularization of broadband networks among these elements, as broadband networks are an important foundation for the development of the information society and digital economy. Therefore, Broadband China Strategy is an essential part of China’s digital infrastructure construction, which plays a key role in digital transformation and has a far-reaching impact on the digital upgrading of China’s economy and the process of social informatization. In summary, BCP, as one of the keys to digital infrastructure construction, and its investigation of green and low-carbon transformation and development of cities is a good complement and improvement to digital infrastructure research, providing valuable experience and inspiration for digital infrastructure construction and sustainable development worldwide.

Taking China as an example, the study provides new ideas and Chinese experience for other countries in strengthening environmental regulation and promoting sustainable development, and supports the potential of digital infrastructure to promote international cooperation, especially in promoting the development of global low-carbon technologies and green finance, which provides a basis for cooperation in the international community’s joint response to climate change. More importantly, the findings of the study support the idea that digital infrastructure development can achieve a win-win situation for both environmental protection and economic growth, which is of strategic importance for other countries to protect the environment while pursuing economic growth.

*2. The logical structure of the paper is unclear, making it challenging to follow. For example, the INTRODUCTION section is excessively lengthy, the METHODS section is too brief, and the discussions are mixed in the RESULTS section. This greatly reduces the readability of this article.*

Response 2: Thank you for your thought-provoking suggestion.

According to your comments, we have made the following modifications. First, we have cut the content of the introduction appropriately. Second, we have adjusted and deepened it to make this part not only more logical, but also closely related to the research topic. Furthermore, we supplement the methods used in this study and present them in the form of a methodological flow. Finally, we rewrote the conclusion to include not only a broad discussion of the findings, but also feasible policy recommendations based on the findings. We believe that the readability of the manuscript can be greatly improved after the above adjustments.

*3. Improvements are needed in the language used throughout the manuscript.*

Response 3: We would like to thank you for kindly reminding us.

We have polished the manuscript to improve the level of language expression, and we have revised, improved and deepened the content of the manuscript. We believe this will improve the readability of the manuscript.

*4. The significance of digital infrastructure is unduly emphasized in the Introduction. It is recommended that the article's focus be shifted to the subject matter at hand as soon as possible. The author's comprehensive introduction provides a detailed account of the Chinese government's stance on digital infrastructure and the initiatives undertaken by China. However, this appears to be tangentially related to the subject matter of the study. It is recommended that the introduction section include more detailed information on the relationship between carbon emissions, digital infrastructure, and economic development. The significance of focusing on the unlocking effect can then be further elucidated by presenting the phenomenon of carbon lock-in.*

Response 4: Thank you for your kind suggestion which is of great help to the integrity of our manuscript.

Based on your suggestions, we have revised the introduction. On the basis of fully introducing the importance of digital infrastructure, we summarize its research progress related to carbon emissions, and further propose the significance of the improvement and layout of digital infrastructure to change the traditional development mode and break the carbon lock-in. We believe that the revised introduction will be more logical, thus increasing the readability of the manuscript. The revisions are as follows here for your convenience.

**L99-121 (Page 5 in all marked versions):**

Many scholars believe that digital infrastructure has the potential to be sustainable and are optimistic about reducing carbon emissions. Romm (2002) found that widespread use of the Internet resulted in a significant reduction in energy intensity in the United States and highlighted the important contribution of ICTs in reducing greenhouse gases. Moyer and Hughes (2012) found that digital infrastructure could reduce global carbon emissions by about 50 years. Liu et al. (2015) verified that digital infrastructure can reduce carbon emissions in China, and there is significant regional heterogeneity in this effect. Asongu et al. (2018) found that digital infrastructure layout has a positive impact on reducing carbon emissions in African countries. Zahra et al. (2019) demonstrate that ICTs can reduce carbon emissions in Iran’s transport sector. Zahra et al. (2019) found that ICT generally reduces carbon emissions in countries along the Belt and Road. On the one hand, digital infrastructure can optimize the industrial structure and promote industrial transformation and upgrading. The reduction of energy use intensity in the service sector helps to reduce carbon emissions. On the other hand, digital infrastructure can promote technological progress, accelerate the research and development and diffusion of innovation, facilitate the development of clean energy, improve energy utilization efficiency, strengthen the monitoring and control of carbon emissions, and thus reduce carbon emissions. It can be seen that digital infrastructure helps to change the traditional development model from relying on large-scale energy consumption to a more low-carbon and sustainable development mode, effectively breaking the carbon lock of traditional industries, and providing key technical and structural support for the global realization of a low-carbon economic path.

*5. The authors wish to discuss a multitude of issues, which could be more effectively addressed by grouping them into two. For instance, the first and fourth issues are, in fact, both concerned with the impact of digital facility construction on carbon lock-in.*

Response 5: We thank you for this careful comment.

Based on your suggestions, we have integrated the research questions to ensure the readability of the manuscript. The revisions are as follows here for your convenience.

**L157-163 (Page 8** **in all marked versions):**

In this regard, China’s digital transformation and its commitment to the “dual carbon” goal make it a good practical case to study the BCP-CLI relationship. Therefore, we cannot help but ask: (1) How does BCP break the China’s CLI? (2) Does BCP have spatial spillover effects and heterogeneity on CLI? The motivation of this study is to answer the above questions and investigate to what extent BCP can influence CLI.

*6. In section 2.2 of LITERATURE REVIEW, the majority of the articles referenced appear to originate from China. It is recommended that further studies from other regions, such as Europe and North America, be cited in order to provide a more comprehensive understanding of the subject matter.*

Response 2: We would like to thank you for this insightful comment.

In response to your comments, we have added the research literature on the relationship between digital development and carbon emissions in Europe and North America in the literature review section to provide a more comprehensive understanding of this topic. The revisions are as follows here for your convenience.

**L249-272 (Page 12 in all marked versions):**

Numerous studies have looked at how digital technologies affect carbon emissions to date (Dong et al. 2022a, Hieu, Le Thanh and Anh 2023, Zhang et al. 2022a, Zhang et al. 2022b), but there is still some controversy. For example, Sadorsky (2012) argues that information and communications technology (ICT) development can raises power usage and carbon emissions. This conclusion is supported by the investigations of Collard, Fève and Portier (2005), who find that ICT do not contribute to improved energy usage and efficiency. Salahuddin and Alam (2016) also fails to identify any benefits of ICT for energy using panel data from OECD countries. Khan (2019), based on data from N-11 countries, found that the use of ICT devices actually increased carbon emissions. However, Lu (2018) found a positive carbon mitigation effect of ICT in 12 Asian countries and considered it as an important strategy to achieve low carbon development. Raheem, Tiwari and Balsalobre-Lorente (2020) demonstrated the G7 countries’ ability to reduce carbon emissions in the long run thanks to ICT. Škare, Gavurova and Porada-Rochon (2024) examines the impact of digitalization on the carbon footprint of governments, households, businesses, NGOs and imports across the EU and draws positive conclusions. Bocean (2023) assesses the positive impact of digital transformation on the economic performance and sustainability of EU countries. Based on data at a global level, Zuo et al. (2024) found that the impact of digital development on carbon reduction is much stronger in Europe and North America than in other countries. In addition, a few scholars believe that the impact of ICT on carbon emissions is not significant. Based on Tunisia’s long time series data, Amri, Zaied and Lahouel (2019) found that ICT has negligible impact on carbon emissions, which means that the Tunisian government can promote the development of ICT.

*7. In the Results section, the authors discuss the spatial spillover effect as one of the topics addressed in their analysis. Nevertheless, this topic is not addressed in the hypothesis section. A discrepancy exists between the content of the hypothesis and the analysis presented in the results section.*

Response 7: Thank you for your helpful comment.

We added the hypothesis that BCP has a spatial spillover effect on CLI (hypothesis 3) to the hypothesis section, so as to ensure that the contents before and after the manuscript correspond. The revisions are as follows here for your convenience.

**L369-387 (Page 17 in all marked versions):**

BCP facilitates the transfer of green technologies, ideas and institutions to neighboring regions (Andreopoulou 2012). Digital technologies can spread to the neighborhood through shared networks and platforms, creating a “spillover effect” that breaks the CLI (Acs et al. 2021). Also, digital technologies enable the public to access information from multiple sources and raise their environmental awareness, thus breaking the CLI (Liu et al. 2022b). The combination of digital technology and government governance has led local governments at all levels to compete to put in place digital governance regulations and systems (Raheem et al. 2020) in order to avoid being eliminated from the new environmentally oriented regional race. Therefore, if the policy is effective, other regions are bound to follow suit. In addition, regions with higher digital technology are more inclined to attract capital intervention, while regions with scarce digital technology will still retain backward productivity, thus reinforcing CLI (Ustundag, Cevikcan and Karacay 2018). This situation also exists in China. Over the past decade or so, there have been serious regional disparities in digital infrastructure (Kane et al. 2017), which in turn has been an important factor in attracting talent and capital (Barata 2019). Thus, the development of BCP can affect CLI through the “siphon effect”. In light of this, we propose the following hypothesis:

Hypothesis 3. BCP has a spatial spillover effect on CLI.

*8. The Methods section does not present the full range of methods employed in the article and the role of these methods in relation to each other. While it is acceptable to detail only the main methods in this section, it is highly recommended that a figure be included to illustrate the methodology of the research.*

Response 8: We would like to thank you for the valuable comment.

In response to your comments, we have drawn a clear methodological flow chart in order to increase the readability of the manuscript. In addition, we reorganize the research theory of the paper and draw the theoretical framework of BCP’s influence on CLI. We believe that your comments will greatly improve the quality of the manuscript. The revisions are as follows here for your convenience.

**L391 (Page 18 in all marked versions):**

Fig. 1 The impact mechanisms of the BCP on CLI.

**L445 (Page 21 in all marked versions):**

Fig.2 Methodological flow.

*9. It is recommended not to mix the results section with the discussion section. The summary in the conclusions section appears somewhat abrupt due to the lack of a detailed discussion of the topic. Furthermore, the policy recommendations lack sufficient support.*

Response 7: We would like to thank you for the valuable comment.

In response to your suggestions, we have re-done the last section to deepen and summarize the content. Specifically, we have discussed the findings extensively and summarized the policy implications. Second, we have organized the policy recommendations and made feasibility suggestions from the research findings, thus enabling the policy recommendations to be supported by the research findings. The revisions are as follows here for your convenience.

**L772-785 (Page 51 in all marked versions):**

With the increasing severity of the global climate change problem and the pursuit of sustainable development, achieving carbon neutrality has become a common challenge faced by various countries. As a policy initiative covering economic, social and environmental dimensions, the impact of BCP on carbon emissions is not only related to the realization of China’s future carbon emission reduction targets, but also provides empirical support and policy reference for global carbon management and climate change governance. By systematically investigating the carbon lock-in effect of BCP, it can provide theoretical support and practical guidance for the formulation and implementation of similar policies globally, and promote the dissemination and implementation of the concept of sustainable development in other countries and regions. Therefore, for the first time, this paper incorporates BCP and CLI into the same theoretical analysis framework and explores their impact mechanisms, synergistic effects and heterogeneity at the city level. The conclusions of the study are as follows:

**L799-817 (Page 52 in all marked versions):**

The findings of this paper add well to the existing literature. In the CLI research, a large body of literature focuses on the ongoing impact of the life cycle of conventional energy infrastructure on carbon emissions. The findings provide new perspectives by revealing how digital infrastructure can break the CLI through technological innovation and industrial structure upgrading. Meanwhile, the results further validate the importance of digital infrastructure in coordinated regional economic development, i.e., BCP is crucial in promoting synergies in inter-regional environmental governance. In addition, the study finds that there is a time lag effect in policy effects, a finding that provides an essential time reference dimension for policy makers and highlights the need to consider medium and long-term impacts when evaluating the effectiveness of BCP implementation. The heterogeneity effect of BCP provides theoretical support for the development of regionally differentiated policies, i.e., the need to take into account local characteristics prior to policy implementation. In summary, this study supplements the research gap between digital infrastructure and carbon lock-in, providing empirical support and scientific basis for policy development. These findings, on the one hand, provide city managers with a more comprehensive and in-depth perspective to help formulate scientific and effective low-carbon development policies, and on the other hand, as the digital transformation deepens, its potential to promote environmentally sustainable development will be further explored and utilized.

**L819-880 (Page 53 in all marked versions):**

In light of the foregoing results, we offer the following policy recommendations. First, the central government should actively promote the construction of digital infrastructure construction, enhance the breadth and depth of regional informatization., Accelerate the penetration of digitalization into traditional industries, strengthen its wide application in various fields such as production and life, and maximize the sharing of digital dividends.

Second, local governments should actively encourage inter-regional exchanges and cooperation in the digital technology and low-carbon green transformation, remove obstacles to the flow of industrial inputs between regions. Optimize the allocation of resources between regions to create regional aggregates with higher levels of digital technology development and higher green economy efficiency. Make full use of the spatial spillover effect of digitalization to create a good demonstration effect among regions.

Third, when planning and designing digital infrastructure, government departments should provide clearer support services for emerging industrial enterprises and accelerate the agglomeration of emerging enterprises, so as to realize the optimization and upgrading of industrial structure and technological innovation. In addition, the knowledge diffusion effect of digital infrastructure can be fully realized through the construction of innovation service platforms and other means.

Fourth, take the policy as an opportunity to increase the government’s investment in the science and technology innovation sector. On the one hand, innovative talents should be cultivated, and on the other hand, intelligent innovation platforms should be established to enhance the positive externalities of scientific and technological innovation capacity in environmental governance. At the same time, actively advocate and strengthen the concept of green production and green consumption, the implementation of strict environmental regulatory policies, forcing enterprises to green low-carbon upgrading, which in turn promotes the energy consumption transformation in the low-carbon direction, and ultimately break the CLI.

Fifth, resource-based cities should make use of digital technological innovation to improve resource efficiency, thereby promoting energy conservation and emission reduction; non-resource-based cities should pay attention to digital infrastructure and promote the optimization and upgrading of industrial structure. Eastern regions should further leverage their digital foundation advantages to promote the research, development and application of low-carbon technologies; while central and western regions should further improve their digital infrastructures to enhance the quality and efficiency of the regional economy. For cities with better digital infrastructure, governments should focus on promoting technological innovation and achieving efficient use of energy and resources; for cities with poorer digital infrastructure, governments need to invest more in upgrading infrastructure and encouraging enterprises and individuals to adopt digital means of production and life.

**We would like to thank you for your professional review work, constructive comments, and valuable suggestions on our manuscript.**

**Reviewer # 2 comment:**

*1. Some sentences are lengthy and contain multiple ideas. It’s recommended to break them into shorter sentences to enhance readability or consider professional proofreading. For instance, “The aim of the research is to statistically assess the effects of BCP on CLI and its mechanisms.” could be simplified to “This research aims to statistically assess the effects and mechanisms of BCP on CLI.”*

Response 1: Thank you for your kind suggestion which is of great help to the readability of our manuscript.

We have fully considered your suggestion. After carefully examining every sentence in the manuscript, we made changes to some of the longer and unclear sentences to ensure the manuscript’s readability. The revisions are as follows here for your convenience.

**L31-39 (Page 2 in all marked versions):**

The digital wave is driving the fourth industrial revolution in the world (Schwab 2017, Yang et al. 2022). Currently, digital development plays an important role in promoting supply-side reform, innovation-driven and structural adjustment, so it has become the key to the implementation of China’s national strategy (Hong, Shi and Zheng 2023, Liu et al. 2022b).

**L49-55 (Page 3 in all marked versions):**

In addition, the digital infrastructure realizes the linkage of environmental information and resource sharing, making its role in environmental governance and supervision increasing prominent (Liu et al. 2018, Niu et al. 2013, Zhang et al. 2019).

**L66-68 (Page 4 in all marked versions):**

Specifically, since 2014, China’s Ministry of Industry and Information Technology has issued a “Broadband China” policy (BCP), and has approved a total of three batches of pilot cities to promote information infrastructure construction in 2015 and 2016.

**L79-82 (Page 4 in all marked versions):**

In particular, China’s National Development and Reform Commission has identified seven major emerging infrastructure projects in 2020, all of which are closely related to the Broadband China strategy.

**L153-154 (Page 7 in all marked versions):**

Based on the above analysis, we infer that the BCP may have an impact on CLI. Moreover, China’s ever-improving digital infrastructure is dramatically changing its development model.

**L207-210 (Page 10 in all marked versions):**

The World Resources Institute (WRI) estimates that infrastructure and equipment have an average lifespan of 27.5 years, but coal plants lasting even longer than 40 years, and their carbon emissions over the entire life cycle are 68 times that of nuclear power plants.

**L301-303 (Page 14 in all marked versions):**

The continuous improvement of digital technology has laid the foundation for the city’s digital transformation, which can enhance the level of intelligent manufacturing and promote green and low-carbon development.

**L316-319 (Page 15 in all marked versions):**

The openness, interactivity and effectiveness of the digital network make up for the deficiencies of the government in environmental management and promote the modernization of environmental governance (Gelenbe and Caseau 2015, Wu et al. 2021a).

**L326-328 (Page 15 in all marked versions):**

In addition, digital technology has given birth to the intelligence of social life. The home office model reduces energy consumption, and intelligent logistics improves supply chain’s efficiency, thus solving institutional lock and social lock (Wu et al. 2021b).

**L334-337 (Page 16 in all marked versions):**

Digital technology plays a pivotal role in regional innovation (Ren et al. 2021). First, digital technology enables a high degree of integration of information after processing, thus optimizing the whole innovation activities process.

**L353-356 (Page 17 in all marked versions):**

For example, the intelligent transformation of the service industry can accelerate the formation of a new industrial production service system and realize the transformation of the traditional consumption structure to green and low-carbon consumption.

**L395-397 (Page 19 in all marked versions):**

However, using the traditional DID method alone to measure the impact of BCP on the CLI is somewhat biased, because there may be some spatial effects.

**L518-520 (Page 29 in all marked versions):**

Therefore, reducing CLI should shift from an economic model with high energy consumption and high carbon emissions to a low-carbon circular development model with high efficiency and high output.

**L587-589 (Page 34 in all marked versions):**

The implementation effect of BCP has a lag. After the implementation of the policy, it does not produce a restraining effect on CLI immediately, but has a significant impact from the fourth year.

**L685-687 (Page 43 in all marked versions):**

BCP makes the proportion of the tertiary industry continue to increase, and can reduce emissions and environmental protection while upgrading and innovating existing products and technologies, thus breaking the CLI.

**L726-736 (Page 47 in all marked versions):**

The reason for this is that the eastern region is economically developed, with better infrastructure and a higher rate of application and penetration of digital technology. This gives the eastern region a greater advantage in the construction and application of broadband networks, and enables it to more effectively utilize digital technologies to enhance the energy efficiency and resource use, and to achieve both productivity and carbon efficiency. In addition, it is easier for the eastern region to obtain policy support and financial investment to promote the green transformation of digital infrastructure. For example, by investing in distributed and centralized renewable energy projects, purchasing renewable energy on a market basis, and subscribing to green power certificates, the eastern region can more effectively reduce carbon emissions from digital infrastructure.

**L772-785 (Page 51 in all marked versions):**

With the increasing severity of the global climate change problem and the pursuit of sustainable development, achieving carbon neutrality has become a common challenge faced by various countries. As a policy initiative covering economic, social and environmental dimensions, the impact of BCP on carbon emissions is not only related to the realization of China’s future carbon emission reduction targets, but also provides empirical support and policy reference for global carbon management and climate change governance. By systematically investigating the carbon lock-in effect of BCP, it can provide theoretical support and practical guidance for the formulation and implementation of similar policies globally, and promote the dissemination and implementation of the concept of sustainable development in other countries and regions. Therefore, for the first time, this paper incorporates BCP and CLI into the same theoretical analysis framework and explores their impact mechanisms, synergistic effects and heterogeneity at the city level. The conclusions of the study are as follows:

**L799-817 (Page 52 in all marked versions):**

The findings of this paper add well to the existing literature. In the CLI research, a large body of literature focuses on the ongoing impact of the life cycle of conventional energy infrastructure on carbon emissions. The findings provide new perspectives by revealing how digital infrastructure can break the CLI through technological innovation and industrial structure upgrading. Meanwhile, the results further validate the importance of digital infrastructure in coordinated regional economic development, i.e., BCP is crucial in promoting synergies in inter-regional environmental governance. In addition, the study finds that there is a time lag effect in policy effects, a finding that provides an essential time reference dimension for policy makers and highlights the need to consider medium and long-term impacts when evaluating the effectiveness of BCP implementation. The heterogeneity effect of BCP provides theoretical support for the development of regionally differentiated policies, i.e., the need to take into account local characteristics prior to policy implementation. In summary, this study supplements the research gap between digital infrastructure and carbon lock-in, providing empirical support and scientific basis for policy development. These findings, on the one hand, provide city managers with a more comprehensive and in-depth perspective to help formulate scientific and effective low-carbon development policies, and on the other hand, as the digital transformation deepens, its potential to promote environmentally sustainable development will be further explored and utilized.

**L822-824 (Page 53 in all marked versions):**

Accelerate the penetration of digitalization into traditional industries, strengthen its wide application in various fields such as production and life, and maximize the sharing of digital dividends.

**L830-833 (Page 53 in all marked versions):**

Optimize the allocation of resources between regions to create regional aggregates with higher levels of digital technology development and higher green economy efficiency. Make full use of the spatial spillover effect of digitalization to create a good demonstration effect among regions.

**L838-843 (Page 53 in all marked versions):**

Third, when planning and designing digital infrastructure, government departments should provide clearer support services for emerging industrial enterprises and accelerate the agglomeration of emerging enterprises, so as to realize the optimization and upgrading of industrial structure and technological innovation. In addition, the knowledge diffusion effect of digital infrastructure can be fully realized through the construction of innovation service platforms and other means.

**L851-859 (Page 54 in all marked versions):**

Fourth, take the policy as an opportunity to increase the government’s investment in the science and technology innovation sector. On the one hand, innovative talents should be cultivated, and on the other hand, intelligent innovation platforms should be established to enhance the positive externalities of scientific and technological innovation capacity in environmental governance. At the same time, actively advocate and strengthen the concept of green production and green consumption, the implementation of strict environmental regulatory policies, forcing enterprises to green low-carbon upgrading, which in turn promotes the energy consumption transformation in the low-carbon direction, and ultimately break the CLI.

**L869-880 (Page 55 in all marked versions):**

Fifth, resource-based cities should make use of digital technological innovation to improve resource efficiency, thereby promoting energy conservation and emission reduction; non-resource-based cities should pay attention to digital infrastructure and promote the optimization and upgrading of industrial structure. Eastern regions should further leverage their digital foundation advantages to promote the research, development and application of low-carbon technologies; while central and western regions should further improve their digital infrastructures to enhance the quality and efficiency of the regional economy. For cities with better digital infrastructure, governments should focus on promoting technological innovation and achieving efficient use of energy and resources; for cities with poorer digital infrastructure, governments need to invest more in upgrading infrastructure and encouraging enterprises and individuals to adopt digital means of production and life.

*2. The introduction in Chapter 1 and the conclusion section in Chapter 6 lack clear statements regarding the research's contributions, significance, and motivation.*

Response 2: We would like to thank you for this insightful comment.

According to your comments, we have clearly explained the significance and contribution of the research in the introduction and conclusion, and rewritten the content of this part. The revisions are as follows here for your convenience.

**L164-184 (Page 8 in all marked versions):**

The following are some contributions: First, we constructed an indicator evaluation system for CLI at the city level and measured and analyzed it spatially and temporally. Second, we integrate BCP and CLI into the same theoretical analysis framework for the first time, and explore the impact and mechanism of BCP on CLI. This helps accelerate the urban carbon neutrality process and provides decision support for solving the dilemma of urban economic development and environmental protection. Furthermore, this study reveals the spatial spillover effect of BCP on CLI, providing a new strategy for regional synergistic emission reduction and environmental governance. Finally, this study explores the heterogeneous effects of BCP on CLI, which will help the central government to continuously improve the relevant policies and develop a more precise and effective CLI multi-governance system in a targeted manner.

**L772-785 (Page 51 in all marked versions):**

With the increasing severity of the global climate change problem and the pursuit of sustainable development, achieving carbon neutrality has become a common challenge faced by various countries. As a policy initiative covering economic, social and environmental dimensions, the impact of BCP on carbon emissions is not only related to the realization of China’s future carbon emission reduction targets, but also provides empirical support and policy reference for global carbon management and climate change governance. By systematically investigating the carbon lock-in effect of BCP, it can provide theoretical support and practical guidance for the formulation and implementation of similar policies globally, and promote the dissemination and implementation of the concept of sustainable development in other countries and regions. Therefore, for the first time, this paper incorporates BCP and CLI into the same theoretical analysis framework and explores their impact mechanisms, synergistic effects and heterogeneity at the city level. The conclusions of the study are as follows:

*3. In Fig. 1, the mechanism diagram illustrating how BCP influences CLI serves as the basis for the experimental section of the article, but it seems to lack explanations for the content of the figure (steps, groups, arrows, and the meaning and sources of each element). It's advisable to clarify the logic and associations with the four indicators quantifying CLI in Section 3.2, enhancing the article's theoretical logic and reliability.*

Response 3: We would like to thank you for the constructive comment.

We redraw Fig. 1 to further clarify the logic and correlation with the quantitative indicators of CLI on the basis of highlighting and explaining the mechanism of BCP to CLI, so as to ensure the theoretical logic and reliability. The revisions are as follows here for your convenience.

**L391 (Page 19 in all marked versions):**

Fig. 1 The impact mechanisms of the BCP on CLI.

*4. In Section 5.3, the heterogeneous analysis, it's essential to clearly describe the classification criteria first. Explain how the sample cities are categorized, including criteria such as resource endowment, geographical location, and digital infrastructure. This will aid readers in understanding the research's methodology and logic, ensuring the interpretability of the results. Furthermore, elaborating on the practical implications of the heterogeneous results is suggested. While the author mentions the policy effects on different categories of cities in the results section, further explanation of the practical implications of these findings, possibly in the subsequent policy recommendations, is advisable.*

Response 4: We would like to thank you for pointing out this issue.

In the heterogeneity analysis of manuscripts, we supplement and summarize the criteria for classifying sample cities, so as to ensure readers’ understanding of the research methodology and logic. In addition, we further explain the practical implications of the above heterogeneity analysis in our policy recommendations. The revisions are as follows here for your convenience.

**L697-701 (Page 45 in all marked versions):**

According to the National Sustainable Development Plan for Resource Based Cities (2013–2020), we classify all the sample cities into two categories, i.e., resource cities and non-resource cities, and estimate the policy effects separately, and the outcomes are displayed in Table 11.

**L718-723 (Page 46 in all marked versions):**

Geographical disparities in China’s enormous nation have an impact on the degree of economic growth among cities. Therefore, according to the classification of each province by the National Bureau of Statistics and the region to which it belongs, we divide the sample cities into two categories based on their geographic locations, i.e., cities in the central and western regions and cities in the eastern regions, then estimate the policy effects separately, and the results are shown in Table 12.

**L750-756 (Page 49 in all marked versions):**

The number of urban Internet broadband access users can reflect the coverage and access of a region’s information infrastructure, which is one of the important foundations for the development of digital economy. Therefore, according to the median of this indicator, we divided the sample cities into two categories, one is the city with relatively perfect information infrastructure, the other is the city with poor information infrastructure, and estimated the policy effect respectively, as shown in Table 13.

**L876-880 (Page 55 in all marked versions):**

For cities with better digital infrastructure, governments should focus on promoting technological innovation and achieving efficient use of energy and resources; for cities with poorer digital infrastructure, governments need to invest more in upgrading infrastructure and encouraging enterprises and individuals to adopt digital means of production and life.

*5. The conclusion section summarizes the research's purpose, key findings, and policy recommendations. However, it's important to ensure logical coherence and organization. It's suggested to add a discussion section, closely linking each conclusion with corresponding evidence or results, or comparing and echoing existing research, to help readers better understand the research's significant findings.*

Response 5: We would like to thank you for the valuable comment.

In response to your suggestions, we have added a discussion section that highlights the findings and implications of this study. This helps to further assist the reader in understanding the important results of the research. The revisions are as follows here for your convenience.

**L796-817 (Page 52 in all marked versions):**

The findings of this paper add well to the existing literature. In the CLI research, a large body of literature focuses on the ongoing impact of the life cycle of conventional energy infrastructure on carbon emissions. The findings provide new perspectives by revealing how digital infrastructure can break the CLI through technological innovation and industrial structure upgrading. Meanwhile, the results further validate the importance of digital infrastructure in coordinated regional economic development, i.e., BCP is crucial in promoting synergies in inter-regional environmental governance. In addition, the study finds that there is a time lag effect in policy effects, a finding that provides an essential time reference dimension for policy makers and highlights the need to consider medium and long-term impacts when evaluating the effectiveness of BCP implementation. The heterogeneity effect of BCP provides theoretical support for the development of regionally differentiated policies, i.e., the need to take into account local characteristics prior to policy implementation. In summary, this study supplements the research gap between digital infrastructure and carbon lock-in, providing empirical support and scientific basis for policy development. These findings, on the one hand, provide city managers with a more comprehensive and in-depth perspective to help formulate scientific and effective low-carbon development policies, and on the other hand, as the digital transformation deepens, its potential to promote environmentally sustainable development will be further explored and utilized.

*6. For future prospects, it's recommended to provide anticipated results and implications, guiding and inspiring more scholars to conduct further research.*

Response 6: We would like to thank you for the valuable comment.

At the end of the manuscript, we put forward the prospect of future research and provide actionable research progress, hoping to inspire more scholars to carry out further research. The revisions are as follows here for your convenience.

**L883-894 (Page 56 in all marked versions):**

Future research needs to be carried out from the following aspects: First, a more refined carbon lock-in index system needs to be developed. Combined with regional and industry characteristics, the extent of carbon lock-in effect and its influencing factors should be assessed. Secondly, the case studies of different regions are integrated to deeply discuss the implementation effect of BCP in different cities and analyze the feasibility and necessity of policy adjustment. Furthermore, a comprehensive framework between digital economy and sustainable development should be constructed, the paths of synergistic development of the two mentioned above should be explored, and targeted policy recommendations should be put forward. Finally, a cross-country comparison is conducted to analyze the differentiated impact of digital development on carbon lock-in in different national contexts.

**We would like to thank you for your professional review work, constructive comments, and valuable suggestions on our manuscript.**
