# Supplementary material for: The urban carbon unlocking effect of digital infrastructure construction: A spatial difference-in-difference analysis from “Broadband China” pilot policy
Source: PLoS One. 2025 Jan 6;20(1):e0316202. doi: 10.1371/journal.pone.0316202 (PMC11703053; doi:10.1371/journal.pone.0316202)
Supplement: Attachment — Submitted filename: Response to Reviewers_R2.docx [file pone.0316202.s002.docx]

**Response to Reviewers Comments**

Dear editor and reviewers:

On behalf of my co-authors, we thank you very much for your letter and for the reviewers’ comments concerning our manuscript entitled “**The urban carbon unlocking effect of digital infrastructure construction: A spatial difference-in-difference analysis from China (PONE-D-24-09583R1)**”. Those comments are all valuable and very helpful for revising and improving our paper, as well as the important guiding significance to our research. We have studied the comments carefully and have made a major revision and re-plan which we hope to meet with the approval. We tried our best to improve the manuscript and all changes made in the revised manuscript are **highlighted in blue** that will not influence the content and the framework of the paper. The points-by-points respond to reviewers’ comments are as follows:

**Reviewer # 1 comment:**

*1. This manuscript investigated the carbon lock-in phenomenon in the economic development of modern countries and how digital infrastructure construction addresses this issue. Taking the “Broadband China” policy as an example, this manuscript raises an interesting topic, which is of great significance to countries seeking structural transformation of their economies, especially developing countries like China.*

*However, it appears that the focus of this manuscript may have been misplaced. The manuscript in question examines the impact of BCP on CLI. While this issue is undoubtedly of great importance to China's economic development, such a narrow focus may diminish the academic merit of the study. This study would be more suitable for publication in a Chinese journal unless the authors decide to shift the focus of the study to reveal the impact of digital infrastructure on CLI and explore this phenomenon with China as the study area.*

Response 1: Thank you for your thought-provoking suggestion. We apologize for any confusion caused by the lack of a clear research object in the title.

In this study, we focus on the impact of digital infrastructure construction on CLI and its mechanism, and take broadband China development strategy as a proxy for digital infrastructure construction. There are two main reasons for this choice: First, broadband networks are a core component of digital infrastructure, and by improving the quality and coverage of broadband networks, they provide the necessary basic support for the development, services and applications of the digital economy. Second, the broadband China strategy is an important national policy in the development of the digital economy, emphasizing the construction and upgrading of digital infrastructure. The implementation of this policy has a direct impact on the city’s digitization process and related infrastructure investment. Therefore, we believe that it is reasonable and feasible to take broadband China as a proxy of digital infrastructure construction, and this has been mentioned in many previous literatures, , and we have summarized some of the relevant literatures below.

1. Xiao, X., Liu, C., & Li, S. (2024). How the digital infrastructure construction affects urban carbon emissions—A quasi-natural experiment from the “Broadband China” policy. Science of The Total Environment, 912, 169284.

2. Li, X., Yang, G., Shao, T., Yang, D., & Liu, Z. (2024). Does digital infrastructure promote individual entrepreneurship? Evidence from a quasi-natural experiment on the “Broadband China” strategy. Technological Forecasting and Social Change, 206, 123555.

3. Peng, H. R., Ling, K., & Zhang, Y. J. (2024). The carbon emission reduction effect of digital infrastructure development: Evidence from the broadband China policy. Journal of Cleaner Production, 434, 140060.

4. Hong, J., Shi, F., & Zheng, Y. (2023). Does network infrastructure construction reduce energy intensity? Based on the “Broadband China” strategy. Technological Forecasting and Social Change, 190, 122437.

5. Zhou, X., Hu, Q., Luo, H., Hu, Z., & Wen, C. (2024). The impact of digital infrastructure on industrial ecology: Evidence from broadband China strategy. Journal of Cleaner Production, 447, 141589.

6. Li, X., He, P., Liao, H., Liu, J., & Chen, L. (2024). Does network infrastructure construction reduce urban–rural income inequality? Based on the “Broadband China” policy. Technological Forecasting and Social Change, 205, 123486.

7. Wu, W., Wang, S., Jiang, X., & Zhou, J. (2023). Regional digital infrastructure, enterprise digital transformation and entrepreneurial orientation: Empirical evidence based on the broadband China strategy. Information Processing & Management, 60(5), 103419.

8. Hu, J., Zhang, H., & Irfan, M. (2023). How does digital infrastructure construction affect low-carbon development? A multidimensional interpretation of evidence from China. Journal of cleaner production, 396, 136467.

9. Hua, Y., & Zhang, H. (2024). Labour misallocation and digital infrastructure: evidence from a quasi-natural experiment of ‘broadband China strategy’. Applied Economics Letters, 1-7.

10. Liu, Y., Liu, K., Zhang, X., & Guo, Q. (2024). Does digital infrastructure improve public Health? A quasi-natural experiment based on China's Broadband policy. Social Science & Medicine, 344, 116624.

At the same time, our main target is the phenomenon of carbon locking in the context of modern national economic development, with a special focus on the role and potential of digital infrastructure development in addressing this problem. Our research findings hope to find out the great potential of digital infrastructure construction with Broadband China strategy as a proxy to solve the carbon lock-in problem, which can provide practical experience and policy reference for other countries in the world facing similar problems. In fact, many countries have also carried out the construction of digital infrastructure similar to broadband China, such as the United States’ “National broadband Plan”, Singapore’s “smart country”, the United Kingdom’s “all-fiber network construction” digital strategy, Germany’s “broadband network” digital agenda, and India’s “Digital India” based on national fiber network upgrades.

In summary, we believe that it is very meaningful to take broadband China as a specific research object to explore the impact of digital infrastructure construction on CLI. At the same time, your comments still give us a lot of inspiration. We reorganized the title of the manuscript to avoid confusion for the readers. The revisions are as follows here for your convenience.

**L1-3 (Page 1 in all marked versions):**

The urban carbon unlocking effect of digital infrastructure construction: A spatial difference-in-difference analysis from “Broadband China” pilot policy

*2. The introduction should concentrate on the subject matter itself, i.e. on the possible link between digital infrastructure and carbon emissions. It must be presented as a general development issue, rather than as a matter solely pertinent to China. Subsequently, an introduction to China's BCP policy should be provided, accompanied by empirical research findings pertaining to the impact of BCP on CLI.*

*Prior to introducing China's policy, it is essential to elucidate the rationale behind the choice of BCP as a research object.*

*Does this indicate that China is grappling with a more significant CLI challenge?*

*Or is it because China's BCP policy is, to some extent, more representative and can serve as a point of reference for other countries around the world facing similar challenges?*

*This question should be addressed in the introduction..*

Response 2: Thank you for your kind suggestion which is of great help to the integrity of our manuscript.

Based on your comments, we have combed through the introduction and reorganized the content of this section. Specifically, we expand the introduction in the following order. First of all, we present the development context of the current social changes brought about by digitalization. Second, we review current research on the relationship between digital infrastructure development and carbon emissions. Thirdly, we summarize the development of digital infrastructure based on broadband network in other countries and explain the rationality and importance of BCP as an agent of digital infrastructure construction. Fourth, we briefly introduced the CLI. Fifth, we propose the problems and contributions to be solved by this research. The last part is the organization of the manuscript.

We believe that the reorganized introduction structure gives the manuscript a clearer logic and greatly increases its readability. The revisions are as follows here for your convenience.

**L28-132 (Page 1 in all marked versions):**

Currently, human society is experiencing a digital social change based on broadband Internet information, and the digital revolution brought about by the spread of information technology has become an important factor in the economic growth of each country [1]. The digital wave is driving the fourth industrial revolution in the world [2, 3]. Internet information broadband as a digital infrastructure has led to the rise of industrial Internet, cloud computing, artificial intelligence and other technologies, which in turn promotes the green transformation of traditional industries, enables different innovation bodies to share and absorb knowledge with higher efficiency and lower cost, and accelerates green technological innovation in the region through the “spillover effect” [6-9]. In addition, the digital infrastructure realizes the linkage of environmental information and resource sharing, making its role in environmental governance and supervision increasing prominent [10-12]. So, it is foreseeable that digital infrastructure provides a good opportunity for urban low-carbon transformation, industrial upgrading and green development [14, 15].

At the same time, many scholars believe that digital infrastructure has the potential to be sustainable and are optimistic about reducing carbon emissions. Romm [16] found that widespread use of the Internet resulted in a significant reduction in energy intensity in the United States and highlighted the important contribution of ICTs in reducing greenhouse gases. Moyer and Hughes [17] found that digital infrastructure could reduce global carbon emissions by about 50 years. Liu, Cong [18] verified that digital infrastructure can reduce carbon emissions in China, and there is significant regional heterogeneity in this effect. Asongu, Simplice [19] found that digital infrastructure layout has a positive impact on reducing carbon emissions in African countries. Zahra, Dehghan [20] demonstrate that ICTs can reduce carbon emissions in Iran’s transport sector and countries along the Belt and Road. On the one hand, digital infrastructure can optimize industrial structure and promote industrial transformation and upgrading. The reduction of energy use intensity in the service sector helps to reduce carbon emissions. On the other hand, digital infrastructure can promote technological progress, accelerate the research and development and diffusion of innovation, facilitate the development of clean energy, improve energy utilization efficiency, strengthen the monitoring and control of carbon emissions, and thus reduce carbon emissions. It can be seen that digital infrastructure helps to change the traditional development model from relying on large-scale energy consumption to a more low-carbon and sustainable development mode, effectively breaking the carbon lock of traditional industries, and providing key technical and structural support for the global realization of a low-carbon economic path.

Therefore, a growing number of countries are proposing plans to grow their digital infrastructure construction marked by broadband network, such as the United States, United Kingdom, and Japan [21-23]. In addition, the Chinese government has proposed a strategy known as “Information Infrastructure Construction (IIC)”. Specifically, since 2014, China’s Ministry of Industry and Information Technology has issued a “Broadband China” policy (BCP), and has approved a total of three batches of pilot cities to promote information infrastructure construction in 2015 and 2016. The policy mainly includes four contents in the upgrading of broadband users in pilot cities, namely, scale, penetration rate, access capacity and application scope. By then, China will have essentially finished building an accessible and fast internet network infrastructure.

On the one hand, BCP, as a core component of digital infrastructure, provides the necessary basic support for the development, services and applications of the digital economy by improving the quality and coverage of broadband networks. On the other hand, BCP is an important national policy for the development of the digital economy, emphasizing the construction and upgrading of digital infrastructure. The implementation of this policy has a direct impact on the city’s digitization process and related infrastructure investments. Therefore, we believe that it is reasonable and feasible to take BCP as a proxy for digital infrastructure, and it is of great significance. Our main objective is to focus on the phenomenon of CLI in the context of economic development in modern countries, with a particular focus on the role and potential of digital infrastructure development in addressing this problem. Our research results hope to find the positive impact of digital infrastructure construction represented by the Broadband network strategy to solve the CLI problem, and provide practical experience and policy reference for other countries in the world facing similar problems.

Carbon lock-in (CLI) describes a phenomenon in which the fossil fuel-based energy consumption structure under the traditional economic development model cannot be changed in the short term, thus making the economy firmly locked into a carbon-based energy system [24-26]. CLI not only impedes the advancement of low-carbon technology and fosters dependency on development pathways, but also poses a persistent danger to harmony in ecosystems and environmental conservation[27]. Therefore, there is a pressing need to find a solution to the growing CLI, thereby decoupling economic growth from traditional development models.

*3. It appears that CLI is not referenced in the introduction, which is a central concept in this study. It may be advisable for the authors to consider incorporating part of section 2.1 into the introduction. It is recommended that the introduction be shortened, as it is currently too lengthy.*

Response 3: We thank you for this careful comment.

According to your suggestions, we have made the following adjustments to the introduction. First of all, we added a description of the CLI in the introduction to ensure that the content before and after the manuscript is more logical. Secondly, we refined the content of the introduction, highlighted key issues, and appropriately reduced the volume of the introduction. Furthermore, we add to the rationale for BCP as a digital infrastructure building agent. Finally, we detail the goal of the study. On the one hand, China is under pressure from the dual-carbon target, and the central government is struggling to meet the challenges posed by the CLI; On the other hand, BCP is more representative of digital infrastructure, and its positive impact on economic development mode, environmental governance and climate change can provide reference and practical experience for other countries that are carrying out digital construction and facing environmental challenges and sustainable development problems.

*4. It is recommended that section 3.2 be a section of descriptive analysis, with CLI measurement as a separate section.*

*Furthermore, the methodology section should elucidate how certain characteristics of BCP are integrated into the CLI measurement methodology.*

Response 4: We would like to thank you for the valuable comment.

Based on your suggestions, we have made the following changes to this section: First, we present all the variable calculations as a separate section in Section 3.2, so as to guarantee its integrity. Second, we put the descriptive analysis of CLI in Chapter 4 results analysis (Section 4.1) to ensure consistency. Third, we specify how to integrate certain features or influences of BCP into the index system of CLI measures. The revisions are as follows here for your convenience.

**L362-384 (Page 18 in all marked versions):**

The dependent variable in this paper is CLI at the city level. As mentioned earlier, although research on CLI is gradually deepening, there is still no standard of measurement. A part of scholars adopts the indicators of carbon sinks and carbon emissions to measure CLI, but disregard the two most crucial implications, i.e., institution and technology. Therefore, in this paper, drawing on the methods proposed by Niu and Liu (29) and Zhao, Taghizadeh-Hesary (32), we comprehensively quantify the CLI at the city level from four aspects, namely, technology, institution, society and industry, and construct the indicator system as shown in Table 1. Importantly, the metrics of the CLI we built are consistent with the characteristics of the BCP. First, broadband construction promotes the development of the information technology industry, which will affect the structural changes of the secondary industry, especially by improving production efficiency and creating new business models to guide the direction of fixed asset investment, and then affect the industry lock-in. Second, the implementation of BCP will affect the local science and technology expenditure and talent structure, promote local governments to increase investment in science and technology research and development and talent training, and then affect the institutional lock-in. Moreover, the increase in broadband may facilitate the rapid adoption of new technologies (such as cloud computing, big data analytics, etc.) and reduce energy intensity and carbon intensity, thereby affecting technological lock-in. Finally, BCP can improve the accessibility of information and drive changes in social behavior. For example, better connectivity may facilitate remote working and online services, thereby reducing reliance on traditional modes of transport, affecting total ridership and, in turn, social lock-in.

**L418-423 (Page 23 in all marked versions):**

4.1 Descriptive analysis of CLI

We adopted principal component analysis to calculate the CLI levels at the national and prefecture separately from 2006-2019. Fig. 3 depicts the trend of CLI at the national level. Table 2 employed the natural breaks method to categorize the CLI of prefecture-level cities and summarized the cities with the 10 highest CLI values in each category separately.

*5. In conclusion, the study should return to the impact of digital infrastructure on CLI. This could be integrated into the corresponding paragraph on line 654.*

Response 5: Thank you for your helpful comment.

Based on your suggestions, we have reorganized this section to highlight the important impact of digital infrastructure in solving CLI problems, which is the goal of this study. The revisions are as follows here for your convenience.

**L694-705 (Page 43 in all marked versions):**

Digital infrastructure drives green technology innovation, improves resource efficiency while reducing environmental pollution. More importantly, it brings about a change in the way the city develops, altering its development model that is overly reliant on traditional resources, thus breaking the CLI [39, 42, 46, 48]. When the traditional development model continues, the output performance of high pollution, high emission and low efficiency will further aggravate CLI and even affect climate change [24, 25]. Therefore, reducing CLI should shift from an economic model with high energy consumption and high carbon emissions to a low-carbon circular development model with high efficiency and high output. Fortunately, this shift can be realized through the digital development strategy. The advancement of digital technology has been an important catalyst for technological innovation, industrial transformation and consumption upgrading [3, 5, 14, 49].

**L706-734 (Page 44** **in all marked versions):**

This research adds well to existing literature. In the extensive discussion of the influential factors of CLI, the positive effects of digitalization have been overlooked in the literature [30, 36, 39-42]. This findings provide new perspectives by revealing how digital infrastructure can break the CLI through technological innovation and industrial structure upgrading. More importantly, on the one hand, this paper verifies the importance of digital infrastructure in the coordinated development of regional economies, that is, it is crucial in promoting the coordination of inter-regional environmental governance; on the other hand, the policy lag effect found in this study provides an important time reference dimension for policymakers, and also emphasizes the need to consider medium- and long-term impacts when evaluating the implementation effect of such programs. However, the above two key issues have never been discussed in previous studies. Moreover, the heterogeneity effect of the digital infrastructure provides theoretical support for the development of regionally differentiated policies, i.e., the need to take into account local characteristics prior to policy implementation. In summary, this study supplements the research gap between digital infrastructure and carbon lock-in, providing empirical support and scientific basis for policy development. These findings, on the one hand, provide city managers with a more comprehensive and in-depth perspective to help formulate scientific and effective low-carbon development policies, and on the other hand, as the digital transformation deepens, its potential to promote environmentally sustainable development will be further explored and utilized.

**We would like to thank you for your professional review work, constructive comments, and valuable suggestions on our manuscript.**

**Reviewer # 2 comments:**

*1. In view of my reading of your paper, I invite you to address the issues noted below, which are relatively minor but nonetheless essential. Manuscript Formatting: I strongly recommend reviewing the manuscript format to ensure it aligns with PLOS ONE's standards. This includes proper reference citation formats and consistent styling of figures and tables throughout the text.*

Response 1: We would like to thank you for kindly reminding us.

We have carefully read the journal’s submission guidelines and made careful changes to our manuscript format to ensure that it can meet PLOS ONE’s standards. This includes the citation format of the references and the format of the figures and tables in the manuscript.

Due to the large number of revisions, please refer to the revised manuscript with tracked changes for detailed revisions on this issue.

*2. Results Section: This section should objectively present your data and findings without interpretation. The language should be concise and descriptive.*

*Discussion Section: This section is where you should interpret the significance of your results, compare them with existing research, and explore their implications. It allows for more subjective insights and theoretical explanations.*

Response 2: We would like to thank you for this insightful comment.

Based on your comments, we have processed the manuscript as follows: First, in the results section, we have retained only the research results and deleted the interpretation. Second, separate the conclusion from the discussion. In conclusion, we summarize the findings of this study. In the discussion section, we highlight the key points of this study, present contributions to previous literature, and explain the importance and implications of this study, based on an extensive discussion of the findings. The revisions are as follows here for your convenience.

**L366-369 (Page 21 in all marked versions):**

The outcomes in column (5) of Table 5 show that the coefficient of BCP is significantly negative, which indicates that digital development is able to break the CLI. Specifically, compared to non-pilot cities, BCP pilot cities are able to reduce the local CLI by 2.54%. Therefore, hypothesis 1 is verified.

**L602-625 (Page 37 in all marked versions):**

6. Conclusions and discussion

6.1 Conclusions

With the increasing severity of the global climate change problem and the pursuit of sustainable development, achieving carbon neutrality has become a common challenge faced by various countries. As a policy initiative covering economic, social and environmental dimensions, the impact of BCP on carbon emissions is not only related to the realization of China’s future carbon emission reduction targets, but also provides empirical support and policy reference for global carbon management and climate change governance. By systematically investigating the carbon lock-in effect of BCP, it can provide theoretical support and practical guidance for the formulation and implementation of similar policies globally, and promote the dissemination and implementation of the concept of sustainable development in other countries and regions. Therefore, for the first time, this paper incorporates BCP and CLI into the same theoretical analysis framework and explores their impact mechanisms, synergistic effects and heterogeneity at the city level. The conclusions of the study are as follows:

(1) BCP can significantly reduce CLI, and after a number of robust tests, the finding is still valid. More notably, the spatial spillover effect of BCP indicates that its contribution to CLI is significant both in neighboring and local cities.

(2) There is a lag in the policy effect of BCP, i.e., its inhibitory effect on CLI becomes significant only from the third year of policy execution.

(3) The contribution of BCP to CLI is more significant in the eastern region, resource-based cities, and cities with a better digital base.

(4) BCP is able to break CLI by promoting green technological innovation and facilitating industrial structure upgrading.

**L626-667 (Page 38 in all marked versions):**

6.2 Discussion

Digital infrastructure drives green technology innovation, improves resource efficiency while reducing environmental pollution. More importantly, it brings about a change in the way the city develops, altering its development model that is overly reliant on traditional resources, thus breaking the CLI [38, 41, 45, 47]. When the traditional development model is continued, the output performance of high pollution, high emission and low efficiency will further aggravate CLI and even affect climate change [23, 24]. Therefore, reducing CLI should shift from an economic model with high energy consumption and high carbon emissions to a low-carbon circular development model with high efficiency and high output. Fortunately, this shift can be realized through the digital development strategy. The advancement of digital technology has been an important catalyst for technological innovation, industrial transformation and consumption upgrading [3, 5, 14, 48].

This research adds well to existing literature. In the extensive discussion of the influential factors of CLI, the positive effects of digitalization have been overlooked in the literature [30, 36, 39-42]. This findings provide new perspectives by revealing how digital infrastructure can break the CLI through technological innovation and industrial structure upgrading. More importantly, on the one hand, this paper verifies the importance of digital infrastructure in the coordinated development of regional economies, that is, it is crucial in promoting the coordination of inter-regional environmental governance; on the other hand, the policy lag effect found in this study provides an important time reference dimension for policymakers, and also emphasizes the need to consider medium- and long-term impacts when evaluating the implementation effect of such programs. However, the above two key issues have never been discussed in previous studies. Moreover, the heterogeneity effect of the digital infrastructure provides theoretical support for the development of regionally differentiated policies, i.e., the need to take into account local characteristics prior to policy implementation. In summary, this study supplements the research gap between digital infrastructure and carbon lock-in, providing empirical support and scientific basis for policy development. These findings, on the one hand, provide city managers with a more comprehensive and in-depth perspective to help formulate scientific and effective low-carbon development policies, and on the other hand, as the digital transformation deepens, its potential to promote environmentally sustainable development will be further explored and utilized.

**L668-708 (Page 41 in all marked versions):**

7. Policy recommendations

In light of the foregoing results, we offer the following policy recommendations. First, the central government should actively promote the construction of digital infrastructure construction, enhance the breadth and depth of regional informatization. Accelerate the penetration of digitalization into traditional industries, strengthen its wide application in various fields such as production and life, and maximize the sharing of digital dividends.

Second, local governments should actively encourage inter-regional exchanges and cooperation in digital technology and low-carbon green transformation, remove obstacles to the flow of industrial inputs between regions. Optimize the allocation of resources between regions to create regional aggregates with higher levels of digital technology development and higher green economy efficiency. Make full use of the spatial spillover effect of digitalization to create a good demonstration effect among regions.

Third, when planning and designing digital infrastructure, government departments should provide clearer support services for emerging industrial enterprises and accelerate the agglomeration of emerging enterprises, so as to realize the optimization and upgrading of industrial structure and technological innovation. In addition, the knowledge diffusion effect of digital infrastructure can be fully realized through the construction of innovative service platforms and other means.

Fourth, take the policy as an opportunity to increase the government’s investment in the science and technology innovation sector. On the one hand, innovative talents should be cultivated, and on the other hand, intelligent innovation platforms should be established to enhance the positive externalities of scientific and technological innovation capacity in environmental governance. At the same time, actively advocate and strengthen the concept of green production and green consumption, the implementation of strict environmental regulatory policies, forcing enterprises to green low-carbon upgrading, which in turn promotes the energy consumption transformation in the low-carbon direction, and ultimately break the CLI. Fifth, resource-based cities should make use of digital technological innovation to improve resource efficiency, thereby promoting energy conservation and emission reduction; non-resource-based cities should pay attention to digital infrastructure and promote the optimization and upgrading of industrial structure. Eastern regions should further leverage their digital foundation advantages to promote the research, development and application of low-carbon technologies; while central and western regions should further improve their digital infrastructures to enhance the quality and efficiency of the regional economy. For cities with better digital infrastructure, governments should focus on promoting technological innovation and achieving efficient use of energy and resources; for cities with poorer digital infrastructure, governments need to invest more in upgrading infrastructure and encouraging enterprises and individuals to adopt digital means of production and life.

**We would like to thank you for your professional review work, constructive comments, and valuable suggestions on our manuscript.**
