# Supplementary material for: The urban carbon unlocking effect of digital infrastructure construction: A spatial difference-in-difference analysis from “Broadband China” pilot policy
Source: PLoS One. 2025 Jan 6;20(1):e0316202. doi: 10.1371/journal.pone.0316202 (PMC11703053; doi:10.1371/journal.pone.0316202)
Supplement: Attachment — Submitted filename: Response to Reviewers.docx [file pone.0316202.s003.docx]

**Response to Reviewers Comments**

Dear editor and reviewers:

On behalf of my co-authors, we thank you very much for your letter and for the reviewers’ comments concerning our manuscript entitled “**The urban carbon unlocking effect of digital infrastructure construction: A spatial difference-in-difference analysis from “Broadband China” pilot policy (PONE-D-24-09583R2)**”. Those comments are all valuable and very helpful for revising and improving our paper, as well as the important guiding significance to our research. We have studied the comments carefully and have made a major revision and re-plan which we hope to meet with the approval. We tried our best to improve the manuscript, and all changes made in the revised manuscript are **highlighted in blue** that will not influence the content and the framework of the paper. The points-by-points respond to reviewers’ comments are as follows:

**Reviewer # 2 comments:**

*1. Currently, the boundaries between the conclusions and the discussion are not clearly defined. The discussion should be based on the research results, summarizing and interpreting them, while comparing them with existing literature, highlighting the similarities and differences, and exploring the mechanisms behind these differences. Additionally, the discussion should further address the limitations and shortcomings of the study. The conclusion should succinctly summarize the core content of the discussion, revert to the research questions, and emphasize the key findings and academic contributions. I suggest reorganizing Sections 6 and 7 to ensure logical clarity.*

Response 1: Thank you for your thought-provoking suggestion. Based on your comment, we have reorganized the content of sections 6 and 7 to ensure that the logic is clearer. The revisions are as follows here for your convenience.

**L587-650 (Page 39 in all marked versions):**

6.1 Discussion

Our key findings suggest that BCP make a significant contribution to breaking the CLI, which is consistent with existing literature supporting digital infrastructure as a way to reduce carbon emissions. Digital infrastructure drives green technology innovation, improves resource efficiency while reducing environmental pollution. More importantly, it brings about a change in the way the city develops, altering its development model that is overly reliant on traditional resources, thus breaking the CLI [36, 39, 43, 45]. When the traditional development model continues, the output performance of high pollution, high emission and low efficiency will further aggravate CLI and even affect climate change [21, 22]. Therefore, reducing CLI should shift from an economic model with high energy consumption and high carbon emissions to a low-carbon circular development model with high efficiency and high output. Fortunately, this shift can be realized through the digital development strategy.

At the same time, BCP accelerates technological innovation and industrial structure upgrading, thus contributing to carbon unlocking. This research adds well to existing literature. In the extensive discussion of the influential factors of CLI, the positive effects of digitalization have been overlooked in the literature [27, 33, 36-39]. These findings provide new perspectives by revealing how digital infrastructure can break the CLI through technological innovation and industrial structure upgrading. The advancement of digital technology has been an important catalyst for technological innovation, industrial transformation and consumption upgrading [3, 12, 46, 82].

More importantly, on the one hand, our findings verify the importance of digital infrastructure in the coordinated development of regional economies, that is, it is crucial in promoting the coordination of inter-regional environmental governance; on the other hand, the policy lag effect found in this study provides an important time reference dimension for policymakers, and also emphasizes the need to consider medium- and long-term impacts when evaluating the implementation effect of such programs. However, the above two key issues have never been discussed in previous studies.

6.2 Conclusion

Based on an empirical analysis of 266 prefecture-level cities in China from 2006 to 2019, this study explores the effects, mechanisms, synergies and heterogeneity of the BCP on CLI. The results are as follows:

(1) BCP can significantly reduce CLI, and after a number of robust tests, the finding is still valid.

(2) The impact of BCP on CLI has a spatial spillover effect, that is, BCP can contribute to CLI in both neighboring and local cities.

(3) There is a lag in the policy effect of BCP, i.e., its inhibitory effect on CLI becomes significant only from the third year of policy execution.

(4) The contribution of BCP to CLI is more significant in the eastern region, resource-based cities, and cities with a better digital base.

(5) BCP is able to break CLI by promoting green technological innovation and facilitating industrial structure upgrading.

With the increasing severity of the global climate change problem and the pursuit of sustainable development, achieving carbon neutrality has become a common challenge faced by various countries. As a policy initiative covering economic, social and environmental dimensions, the impact of BCP on carbon emissions is not only related to the realization of China’s future carbon emission reduction targets, but also provides empirical support and policy reference for global carbon management and climate change governance. By systematically studying the carbon locking effect of BCP, we can not only provide theoretical support and practical guidance for the formulation and implementation of similar policies worldwide, but also further address global carbon management, so as to solve the environmental challenges brought by climate change and promote the development and realization of sustainable development goals in other countries and regions.

*2. Thank you for adjusting the table formats. However, to enhance the professionalism and consistency of the paper, I recommend unifying the table style (e.g., either use three-line or full-line tables throughout).*

Response 2: Thank you for your kind suggestion. Based on your comment, we have adjusted the format of the form to ensure professionalism and consistency. We used the three-line tabular format to ensure the consistency of the tables in the manuscript. Please refer to the manuscript with all marked versions for detailed revisions.

**We sincerely thank you for your professional review work, constructive comments, and valuable suggestions on our manuscript.**
